# Subcutaneous Tissue Responses to Three Endodontic Irrigants: A Comparative Study

**Published:** 2012-08-01

**Authors:** Kazem Ashofteh Yazdi, Mohammad Sabeti, Pooriya Motahhary, Alireza Kolahdouzan, Mohsen Shayesteh, Noushin Shokouhinejad

**Affiliations:** 1. Dental Research Center, Department of Endodontics, Dental School, Tehran University of Medical Sciences, Tehran, Iran; 2. Department of Endodontics, Dental School, University of Southern California, Los Angeles, California, USA; 3. Department of Oral Pathology, Dental School/Dental Research Center, Tehran University of Medical Sciences, Tehran, Iran; 4. Department of Endodontics, Dental School, Ghazvin University of Medical Sciences, Ghazvin, Iran; 5. Dentist, Tehran, Iran; 6. Iranian Center for Endodontic Research, Shahid Beheshti University of Medical Sciences, Tehran, Iran

**Keywords:** EDTA, Inflammation, MTAD, Sodium hypochlorite, Subcutaneous Tissue, Toxicity

## Abstract

**Introduction:**

This study aimed to compare the subcutaneous tissue responses to MTAD (mixture of a tetracycline isomer, an acid, and a detergent), 17% EDTA, and 2.6% NaOCl.

**Materials and Methods:**

Thirty-six Wistar albino rats were used for this study. Test solutions were injected subcutaneously into predetermined areas on the animal dorsum. The rats were then randomly divided into three groups of twelve each and sacrificed at 2 hours, 2 days, and 2 weeks. The severity of inflammation induced by each irrigant at different time intervals was assessed histologically. The data were analyzed using Kruskal-Wallis and Friedman tests.

**Results:**

The difference in severity of inflammatory reactions induced by tested irrigants at the different time intervals was statically significant (P<0.05). There was no significant difference between the severity of inflammation induced by MTAD and 2.6% NaOCl at the various time intervals (P>0.05). Subcutaneous tissue responses to MTAD were not different from those observed in 17% EDTA specimens at 2-hour and 2-day intervals (P>0.05).

**Conclusion:**

Under the conditions of this study, MTAD has the same toxicity as 2.6% NaOCl.

## Introduction

Removal of bacteria from the root canal system is necessary for successful root canal therapy. Effective endodontic treatment and consequent healing depends on thorough chemomechanical cleaning and shaping of the root canal system [1]. An ideal root canal irrigant should dissolve vital and necrotic tissues in the canal, flush out debris, have antimicrobial effects, and remove the smear layer [2]. Various types of irrigating solutions are available for endodontic use, such as sodium hypochlorite (NaOCl), chlorhexidine (CHX), ethylenediaminetetraacetic acid (EDTA), iodine potassium iodide (IKI), and MTAD (Dentsply Tulsa Dental, Tulsa, OK) [1][3][4][5]. NaOCl is able to dissolve pulpal tissues [6] and act as an antimicrobial agent against most microorganisms and their biofilms [7][8][9]. However, aside from NaOCl unpleasant taste, NaOCl is toxic [10] and does not remove the smear layer [11].

EDTA, however, does remove the mineralized portion of the smear layer [12]. For effective removal of both organic and inorganic components of the smear layer, combined application of NaOCl and a chelating agent, such as EDTA, is recommended [13][14].

MTAD, introduced by Torabinejad in 2003, is an aqueous solution of 3% doxycycline, a broad-spectrum antibiotic; 4.25% citric acid, a demineralizing agent; and 0.5% polysorbate 80 detergent (Tween 80) [15]. MTAD final rinse was shown to effectively remove the smear layer with minimal erosive changes to the surface dentin compared with EDTA [3]. Furthermore, MTAD can eliminate Enterococcus faecalis [16][17][18].

Endodontic irrigants can come in contact with periradicular tissues. Use of toxic irrigants can not only cause complications and tissue damage during root-canal treatment but may also interfere with the repair process [2][19]. Therefore, when choosing irrigants during root-canal treatment low toxicity, high biocompatibility and high efficacy of the irrigants should be considered [19].

Researchers have evaluated the biocompatibility of MTAD and have found that it was less cytotoxic and more biocompatible than other irrigating solutions [20][21][22][23]. MTAD has been reported to exhibit a lower cytotoxicity against L929 cells compared to 5.25% NaOCl, EDTA, and Ca(OH)_2_ paste [20]. In a study by Ring et al. [21], the cytotoxicity of NaOCl/MTAD was reported to be slightly less than NaOCl/EDTA and NaOCl, indicating that MTAD was more biocompatible than NaOCl. The present study aimed to compare the subcutaneous tissue responses to three commonly used endodontic irrigants including MTAD, 17% EDTA, and 2.6% NaOCl.

## Materials and Methods

The research protocol was approved by the research ethics committee of Tehran University. Thirty-six Wistar albino rats weighing 180-220 g were used for this study. Animals were anesthetized with an intramuscular injection of Ketamine hydrochloride 25 to 44 mg/kg and acetylpromazine 0.75 mg/kg. The back of the rats were shaved and disinfected with 70% ethanol. Four circles with 1.5-2 cm distance from each other were drawn on the dermis of the rat by an indelible marker, two on each side of vertebral column.

A total of 0.1 mL of Biopure MTAD (Dentsply Tulsa Dental, Tulsa, OK), 17% EDTA Solution, 2.6% NaOCl, and 0.9% normal saline as control, were injected subcutaneously into the center of each circle in the back of each rat by an insulin syringe. The rats were then randomly divided into three groups of twelve and sacrificed at 2 hours, 2 days, and 2 weeks. Samples of dermis, epidermis and parts of the surrounding muscles were removed and fixed in 10% buffered formalin solution for 2 weeks. 5-µm tissue sections were prepared and stained with haematoxylin and eosin and then evaluated under a light microscope (BX-SI, Olympus, Japan) with different magnifications. A pathologist who was unaware of test groups evaluated the specimens.

According to ISO 7405:1997 standard, the tissue and inflammatory reactions were graded as follows [24]:

1. None: No inflammatory cells infiltration

2. Mild: Scattered chronic inflammatory cells without tissue changes

3. Moderate: Focal inflammatory cell infiltration with tissue changes but without necrosis

4. Severe: Severe infiltration of inflammatory cells

5. Abscess: Abscess formation

The severity of inflammatory reactions induced by irrigants was analyzed using Kruskal-Wallis test. Friedman test was used for analysis of inflammatory reactions of each irrigant at different time intervals. Statistical analysis was performed with SPSS 11.5 for Windows.

## Results

**Figure 1:**
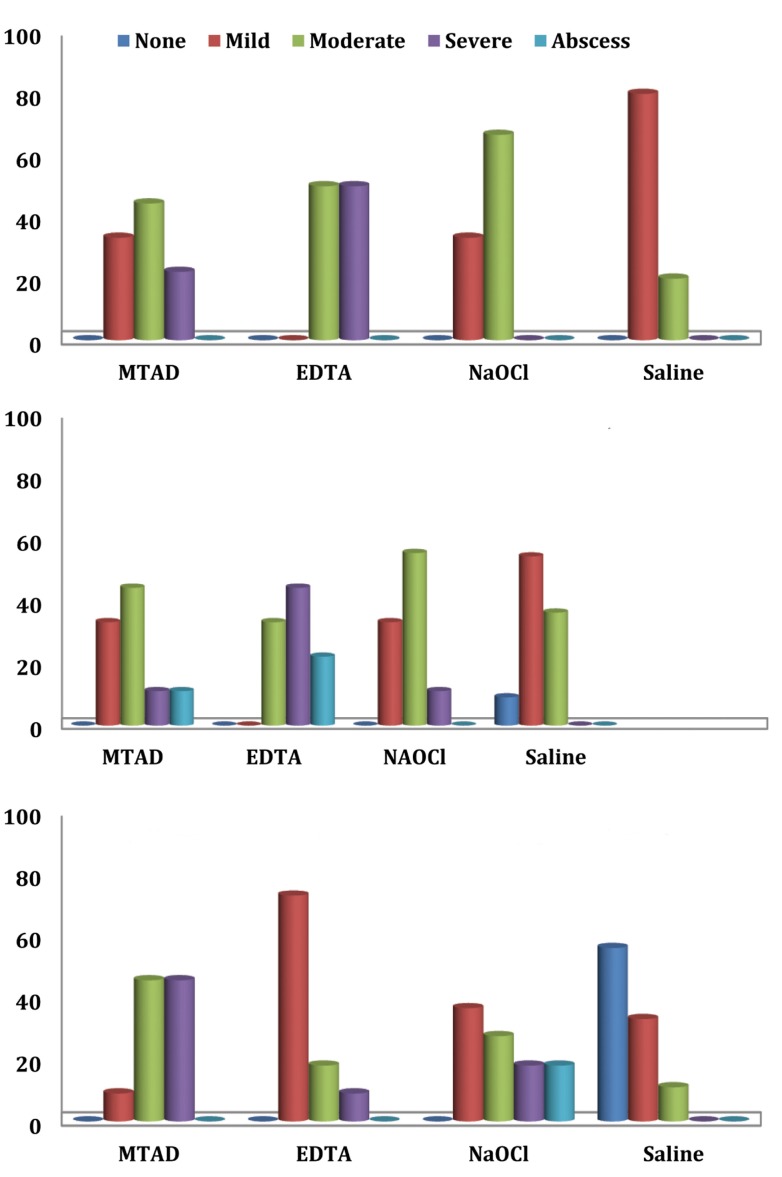
Severity of inflammatory reactions to test irrigants after: A) 2 hours; B) 2 days; C) 2 weeks)

**Figure 2:**
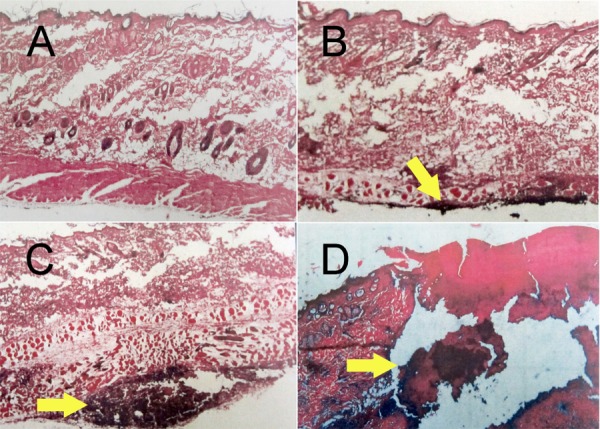
Subcutaneous tissue responses to irrigants: A) No inflammatory cells infiltration; B) Moderate inflammatory cells infiltration with tissue changes but without necrosis; C) Severe infiltration of inflammatory cells; D) Abscess formation

The severity of inflammatory reactions induced by test solutions at different time intervals is shown in Figure 1. The photographs of some subcutaneous tissue responses to test irrigants are presented in Figure 2. The difference in tissue responses to the irrigants at different time intervals was statically significant (P<0.05).

At 2-hour interval, no significant difference was found between the inflammatory reactions induced by MTAD and those induced by 2.6% NaOCl and 17% EDTA (P>0.05). The difference in inflammatory reactions between 17% EDTA and 2.6% NaOCl (P=0.006) and 17% EDTA and normal saline (P<0.001) was statistically significant.

There was no significant difference in the degree of inflammatory reactions induced by tested irrigants at 2-day interval (P=0.061).

The results showed significant difference in the severity of inflammation between four irrigants at 2-week interval (P<0.000). There was a significant difference in the degree of inflammation between MTAD and 17% EDTA (P=0.005), and MTAD and normal saline (P<0.001); however, the difference between MTAD and 2.6% NaOCl was not significant (P=0.562). NaOCl also showed significant difference when compared to normal saline (P<0.001); however 17% EDTA did not (P=0.031).

The results showed no significant differences between the severity of inflammatory reactions induced by MTAD and 2.6% NaOCl at 2-hour, 2-day, and 2-week intervals (P>0.05). The difference between inflammatory reactions of MTAD and 17% EDTA was significant only at 2-week interval (P=0.005).

## Discussion

In the present study, subcutaneous tissue responses to tested endodontic irrigants were evaluated using rats as an in vivo animal model. Yesilsoy et al. used guinea pigs as an in vivo model to examine subcutaneous local tissue reactions to some irrigants [25].

The findings of this study revealed that the severity of inflammation and subcutaneous tissue responses to MTAD and 2.6% NaOCl was not significantly different at any of the time intervals. On the contrary, Zhang et al. showed that the cytotoxicity of MTAD on L929 fibroblasts was more than that of 2.63% NaOCl after 24 hours [20]. They also demonstrated that MTAD was less cytotoxic than EDTA [20]. However, in the present study, the severity of inflammatory reactions induced by MTAD was not different from that induced by EDTA except at the 2-week interval. The findings of the present study also contradict Yasuda et al. who found MTAD to be less cytotoxic on MC3T3-E1 and periodontal ligament cells compared with 5.25% NaOCl and 17% EDTA at 24 hours [23]. These controversial results might be partly attributed to the difference in test subjects which were fibroblast cells in the study by Zhang et al. [20], MC3T3-E1 and periodontal ligament cells in the study by Yasuda et al. [23] and rats’ subcutaneous tissue in the present study.

In contrast to this investigation, Yesilsoy et al. in a study on subcutaneous tissue reactions found that 0.5% and 2.5% NaOCl exhibited no inflammation at 2 hours; however, tested solutions induced mild inflammation after 2 days [25].

The variation between the findings of different studies could be attributed to the concentration of tested solutions. It has been shown a correlation between the cytotoxicity of NaOCl and its concentration [20]. Furthermore, the time intervals and design of investigations might affect the results of various studies.

## Conclusion

Under the conditions of this animal study, MTAD was as cytotoxic as 2.6% NaOCl. The subcutaneous tissue reaction to MTAD was similar to EDTA after 2 hours and 2 days.
